# Routes to diagnosis for hepatocellular carcinoma patients: predictors and associations with treatment and mortality

**DOI:** 10.1038/s41416-024-02645-3

**Published:** 2024-03-18

**Authors:** Anya Burton, Jennifer Wilburn, Robert J. Driver, David Wallace, Sean McPhail, Tim J. S. Cross, Ian A. Rowe, Aileen Marshall

**Affiliations:** 1 National Disease Registration Service, NHS England, Quarry House, Quarry Hill, Leeds, LS2 7UE UK; 2 Bristol Medical School, Canynge Hall, 39 Whatley Road, Bristol, BS8 2PS UK; 3 Leeds Institute for Medical Research at St. James’s, University of Leeds, Leeds, LS9 7TF UK; 4 Department of Health Services Research & Policy, London School of Hygiene & Tropical Medicine, 15-17 Tavistock Place, London, WC1H 9SH UK; 5 Institute of Liver Studies, Kings College Hospital, Denmark Hill, London, SE5 9RS UK; 6 Liverpool Experimental Cancer Medicine Centre, University of Liverpool, Liverpool, L7 8XP UK; 7 Sheila Sherlock Liver Centre, The Royal Free Hospital, London, NW3 2QG UK

**Keywords:** Epidemiology, Gastrointestinal cancer

## Abstract

**Background:**

Hepatocellular carcinoma (HCC) incidence has increased rapidly, and prognosis remains poor. We aimed to explore predictors of routes to diagnosis (RtD), and outcomes, in HCC cases.

**Methods:**

HCC cases diagnosed 2006–2017 were identified from the National Cancer Registration Dataset and linked to Hospital Episode Statistics and the RtD metric. Multivariable logistic regression was used to explore associations between RtD, diagnosis year, 365-day mortality and receipt of potentially curative treatment.

**Results:**

23,555 HCC cases were identified; 36.1% via emergency presentation (EP), 30.2% GP referral (GP), 17.1% outpatient referral, 11.0% two-week wait and 4.6% other/unknown routes. Odds of 365-day mortality was >70% lower via GP or OP routes than EP, and odds of curative treatment 3–4 times higher. Further adjustment for cancer/cirrhosis stage attenuated the associations with curative treatment. People who were older, female, had alcohol-related liver disease, or were more deprived, were at increased risk of an EP. Over time, diagnoses via EP decreased, and via GP increased.

**Conclusions:**

HCC RtD is an important predictor of outcomes. Continuing to reduce EP and increase GP and OP presentations, for example by identifying and regularly monitoring patients at higher risk of HCC, may improve stage at diagnosis and survival.

## Introduction

Cancer survival in the UK is improving but remains poor compared to other European [1] and high income countries [2]. Improving cancer survival in the UK is a key challenge set out by the Department of Health, the NHS and the Independent Cancer Taskforce [3–5]. Survival rates vary greatly between cancers [1]. Primary liver cancer has a particularly poor prognosis compared to many other cancers [1]. Furthermore, incidence increased over three-fold for hepatocellular carcinoma (HCC), the most common form of liver cancer, between 1997 and 2017 [6]. HCC diagnosis is strongly associated with liver cirrhosis such that approximately 80% of patients with HCC also have liver cirrhosis. Early detection at a treatable stage improves cancer prognosis [7]. As such, 6-monthly liver ultrasounds for cancer detections are recommended for people at high risk of HCC [8].

The route a person takes to their diagnosis of cancer is strongly predictive of 1-year survival [9]. The Routes to Diagnosis (RtD) metric uses an algorithm to assess information about interactions with health care prior to a cancer diagnosis to assign each patient to a route [9]. Emergency presentations (via A&E, or emergency GP referral, transfer, consultant outpatient referral, admission or attendance) [9], have the poorest survival for the majority of cancers (https://www.cancerdata.nhs.uk/routestodiagnosis/survival). Conversely, in general, routine and urgent GP referral have the best prognosis. Overall, one in five cancers are diagnosed as an emergency; for HCC it is one in three. Understanding the differences in RtD presentations over time and predictors of RtDs can help us understand the survival gap and indicate ways to address it. Here we aim to explore, in HCC cases for the first time, (1) predictors of RtD, (2) differences in RtD presentations over time, (3) associations with mortality, and (4) associations with receipt of potentially curative treatment.

## Methods

### Registry data

Patient-level data on England residents diagnosed with HCC (defined as 10th edition of the International Classification of Diseases (ICD-10) code C22.0 and the second edition ICD Oncology (ICD-O) morphology code M8170) between 1st January 2006 and 31st December 2017 were extracted from the National Cancer Registration Dataset [10]. The Registration Dataset uses data from a wide range of sources including hospital activity records, multidisciplinary teams meetings, patient administration systems, pathology reports, molecular test reports and death certificates from the Office for National Statistics. It includes all cases of cancer diagnosed and treated in the National Health Service (NHS) in England (which funds 98–99% of all hospital activity [11]), as well as some treated privately [10]. A marker of registration quality is the proportion of cancers identified through death certification alone. In the English National Cancer Registration Database this is less than 1%, indicating that the vast majority of data relevant to a cancer diagnosis is being captured and the cancer registry has very high population completeness [10].

If a patient had two HCC tumours diagnosed during the study period, only the first tumour was included. Only patients aged 20 or over were included, due to rare aetiologies and subtypes of HCC in young people (47 aged <20 years excluded). HCC diagnoses were based on clinical investigations (imaging) in 60% of cases (as recommended for cirrhotic patients by EASL [12]), pathology in 35% of cases, and death certificate only or unknown in the remaining 5%. Data extracted included diagnosis date, death date, vital status (alive/dead/emigrated), date of last vital status (follow-up to 02/03/2020), age at diagnosis, gender, ethnicity (broad groups) and Charlson comorbidity score (categorical: no known comorbidities, 1, 2, 3 or more) and index of multiple (IMD) deprivation quintile (depending on year of diagnosis, income domains 2007, 2010, 2015, or 2019 as measured for each lower super output area (administrative areas of approximately 400–1200 households) was used, linked via patient postal address code at diagnosis [13]). Data on these variables was complete except for 3 people in which a Charlson score had not been derived.

### Hospital episode statistics

These data were linked to the Hospital Episode Statistics Admitted Patient Care dataset (HES APC) [14]. HES data were used to identify cirrhosis status and underlying primary liver disease: cirrhosis status was defined as either compensated, decompensated, or none/unknown based on relevant diagnostic and therapeutic codes, as detailed in Driver et al. [15] and in the supplementary information. Underlying cause of primary liver disease (PLD) was assigned based on diagnostic codes recorded between five years before and one year after diagnosis. Including diagnostic codes up to one year after diagnosis showed improved PLD identification (supplementary information). The following hierarchy was applied based on relative risk of HCC associated with each PLD to give one primary PLD per patient [16]: hepatitis C (HCV) > hepatitis B (HBV) > primary biliary cirrhosis (PBC) > autoimmune hepatitis > haemochromatosis > alcohol-related liver disease (ALD) > non-alcoholic fatty liver disease (NAFLD). NAFLD was assigned when a patient had fatty (change of) liver, not elsewhere classified, or cirrhosis combined with obesity or diabetes, without any other PLD. Those with no relevant diagnostic codes were designated as ‘Other/unknown’.

### Treatment

Two sources were used to derive receipt of potentially curative treatment: HES APC records [14] and the Registration Dataset treatment data [10]. Treatments given from 60 days before diagnosis until death or 2 years (730 days) after diagnosis were included. Pre-diagnosis treatment was included as HCC may be diagnosed clinically and treated prior to the official registry date of incidence which may take histological tissue as the gold standard for diagnosis and reassign date of diagnosis once tissue is received [17]. The most definitive HCC treatment a patient received during this time was captured based on the following hierarchy of potentially curable treatments: liver transplant > liver resection > radiofrequency or microwave ablation > irreversible electroporation > percutaneous ethanol injection.

### Route to diagnosis

Each HCC case was assigned a route to diagnosis (RtD). Details of methods used to identify and assign these are given in Elliss-Brookes et al. [9]. In brief, the algorithm interrogates cancer registration data linked to routine data from immediately prior to diagnosis (including HES, Cancer Wait Times, and NHS Screening programme data), and assigns a main RtD category: emergency presentation (EP), general practice (GP) referral, Two Week Wait (TWW, urgent GP referral with a suspicion of cancer), inpatient elective (IP), other outpatient (OP), death certification only (DCO, no data available other than a death certificate flagged by the registry) and Unknown. The methodology was developed using data for all patients with newly diagnosed malignant cancer registered in the English National Cancer Registration system between 2006 and 2008 (739,667 tumours). Since then, the metric has been derived yearly for all cancer new registrations and forms part of the cancer registration dataset.

### Patient and public involvement statement

No patients were involved in forming the research question or selecting the outcome measures, nor were they involved in developing the study design. No patients were asked to advise on interpretation or writing up of results. Results will be shared through patient charities, regionally and nationally, including the British Liver Trust and on the NDRS and British Association for the Study of the Liver websites.

## Statistical methods

### Descriptive

The distribution of demographic and clinical factors in cases by RtD were described using means and standard deviation (for normally distributed continuous data), medians and IQRs (skewed continuous data) and absolute numbers and percentages (categorical data).

### Associations with RtD and differences over time

Associations of the four main HCC routes to diagnosis (EP, OP, TWW and GP) with demographic and clinical variables (year of diagnosis, age, person stated gender, area-based deprivation quintile, ethnicity, Charlson comorbidity score, cirrhosis stage, PLD) were examined using multivariable logistic regression models. Unknown, DCO and IP were not examined due to smaller numbers. Proportions presenting via each route by year of diagnosis, unadjusted, demographic-adjusted (age, gender, ethnicity and deprivation quintile) and fully-adjusted (demographic variables and Charlson comorbidity score, cirrhosis stage, primary liver disease) were calculated.

### Association with curative treatment

Associations of RtD with receipt of potentially curative treatment, unadjusted, and adjusted for multiple factors (age, gender, diagnosis year, deprivation quintile, ethnicity, Charlson comorbidity score, cirrhosis stage, and PLD) were assessed using logistic regression to calculate odds ratios (ORs). Those with DCO as the RtD were excluded from these analyses as none received curative treatment (*n* = 92). Sensitivity analyses were conducted excluding those that died within 90 days of diagnosis, adjusted for TNM stage (not included in the main model as stage was only available for 27% of cases), and stratified by cirrhosis status, age (< or ≥65years), and PLD.

### Mortality

Those with vital status uncertainty due to discrepancies with recorded date of death (date of death before date of diagnosis (*n* = 9), date of death before date of treatment (*n* = 15), patient traced alive after date of death (*n* = 11)) and those lost to follow up (i.e. emigrated (*n* = 50), or with missing vital status (*n* = 5), were excluded. Those with DCO as the RtD were also excluded (*n* = 92) as there was no variation in survival in this group (date of death and date of diagnosis were the same). This left 23,373 individuals for mortality analysis. Univariable and multivariable logistic regression (adjusted for age, gender, diagnosis year, deprivation quintile, ethnicity, Charlson comorbidity score, cirrhosis status, and PLD) were used to calculate odds of 90-day and 365-day (one-year) mortality from diagnosis date, by RtD. Sensitivity analyses were conducted adjusting for TNM stage, and additionally for receipt of curative treatment, and stratified by cirrhosis status, age (< or ≥ 65years), and PLD.

Model assumptions were checked and met for all analyses performed. Analyses were conducted using Stata version 17.1 StataCorp. 2017. *Stata Statistical Software: Release 17*. College Station, TX: StataCorp LLC.

## Results

### Descriptive

Overall, in England 23,555 HCCs were diagnosed between 2006 and 2017. 36.1% of these presented via an emergency route, 30.2% via GP referral, 17.1% via Outpatient referral and 11.0% via Two Week Wait, 1.3% Inpatient Elective, 0.4% DCO and 3.9% had no data available (‘Unknown’ route) (Table 1).

**Table 1:** Description of HCC cases by route to diagnosis.

	Overall	Emergency	GP referral	Outpatient	Two week wait	Inpatient	DCO	Unknown
No. of patients	23555	36.1% (8492)	30.2% (7107)	17.1% (4037)	11.0% (2583)	1.3% (315)	0.4% (92)	3.9% (929)
Mean age (years)	69.1	70.2	68.5	66.4	71.9	66.0	69.4	67.4
Male gender	78.1% (18402)	75.9% (6448)	79.5% (5651)	79.0% (3188)	79.6% (2056)	81.3% (256)	76.1% (70)	78.9% (733)
Median survival (days)^a^	194	55	401	491	204	192	-	205
Receipt of curative treatment	20.5% (4832)	9.0% (764)	27.8% (1978)	35.8% (1445)	15.9% (410)	27.6% (87)	0.0% (0)	15.9% (148)
Deprivation quintile								
1 – least	15.6% (3665)	13.8% (1173)	16.1% (1142)	16.4% (661)	16.8% (433)	19.4% (61)	20.7% (19)	18.9% (176)
2	17.9% (4212)	16.6% (1406)	18.5% (1315)	17.6% (709)	19.7% (508)	21.3% (67)	15.2% (14)	20.8% (193)
3	19.8% (4655)	19.5% (1652)	20.0% (1419)	20.0% (806)	20.8% (538)	19.0% (60)	25.0% (23)	16.9% (157)
4	21.4% (5039)	22.6% (1917)	20.3% (1440)	21.8% (880)	21.0% (543)	17.8% (56)	15.2% (14)	20.3% (189)
5 - most	25.4% (5984)	27.6% (2344)	25.2% (1791)	24.3% (981)	21.7% (561)	22.5% (71)	23.9% (22)	23.0% (214)
Ethnicity								
White	83.5% (19667)	83.5% (7093)	85.2% (6058)	82.7% (3340)	87.2% (2253)	77.5% (244)	35.9% (33)	69.5% (646)
Black	2.8% (666)	2.6% (224)	2.8% (199)	3.4% (136)	2.1% (53)	6.7% (21)	1.1% (1)	3.4% (32)
South Asian	4.7% (1096)	5.0% (423)	4.7% (336)	5.2% (209)	3.1% (79)	4.4% (14)	2.2% (2)	3.6% (33)
Other Asian	2.3% (543)	2.1% (180)	2.3% (162)	3.1% (127)	1.7% (45)	1.3% (4)	2.2% (2)	2.5% (23)
Other ethnicity	1.8% (414)	3.1% (266)	1.5% (104)	2.5% (101)	1.3% (33)	2.5% (8)	2.2% (2)	2.4% (22)
Not known	5.0% (1169)	4.9% (417)	3.5% (248)	3.1% (124)	4.6% (120)	7.6% (24)	56.5% (52)	19.8% (184)
Primary liver disease								
Autoimmune hepatitis	1.0% (243)	0.9% (79)	1.3% (91)	1.5% (62)	0.2% (5)	0.3% (1)	0.0% (0)	0.5% (5)
Alcohol-related liver disease	20.7% (4871)	24.0% (2037)	20.0% (1418)	21.5% (868)	12.8% (331)	19.7% (62)	10.9% (10)	15.6% (145)
Haemochromatosis	2.9% (672)	1.9% (159)	3.6% (254)	4.4% (176)	2.1% (54)	2.9% (9)	0.0% (0)	2.2% (20)
Hepatitis B	3.6% (847)	2.9% (249)	3.9% (278)	5.7% (232)	1.8% (46)	6.0% (19)	0.0% (0)	2.5% (23)
Hepatitis C	13.2% (3109)	11.6% (987)	15.1% (1076)	19.3% (781)	4.6% (120)	11.4% (36)	4.3% (4)	11.3% (105)
Non-alcoholic fatty liver disease	15.1% (3557)	15.3% (1301)	16.6% (1181)	14.4% (582)	14.1% (363)	14.0% (44)	2.2% (2)	9.0% (84)
Primary biliary cirrhosis	2.0% (478)	1.8% (155)	2.0% (144)	3.4% (138)	0.7% (18)	1.0% (3)	2.2% (2)	1.9% (18)
Other/unknown/none	41.5% (9778)	41.5% (3525)	37.5% (2665)	29.7% (1198)	63.7% (1646)	44.8% (141)	80.4% (74)	56.9% (529)
Co-morbidities								
None	41.7% (9833)	41.5% (3520)	39.2% (2783)	30.8% (1242)	57.2% (1477)	55.9% (176)	69.6% (64)	61.5% (571)
One	17.5% (4122)	17.0% (1441)	18.2% (1294)	17.2% (694)	20.3% (524)	17.8% (56)	16.3% (15)	10.5% (98)
Two	11.0% (2593)	10.8% (917)	11.5% (815)	12.2% (493)	10.3% (266)	6.7% (21)	3.3% (3)	8.4% (78)
Three or more	29.7% (7004)	30.8% (2613)	31.2% (2214)	39.8% (1607)	12.2% (316)	19.7% (62)	10.9% (10)	19.6% (182)
Cirrhosis stage								
No cirrhosis/unknown	42.8% (10083)	38.8% (3297)	41.3% (2932)	34.0% (1371)	65.8% (1700)	47.6% (150)	87.0% (80)	59.5% (553)
Compensated cirrhosis	32.5% (7666)	22.0% (1871)	41.7% (2964)	44.7% (1805)	25.4% (657)	29.2% (92)	8.7% (8)	29.0% (269)
Decompensated cirrhosis	24.7% (5806)	39.1% (3324)	17.0% (1211)	21.3% (861)	8.7% (226)	23.2% (73)	4.3% (4)	11.5% (107)
TNM stage								
1	5.4% (1268)	3.1% (263)	7.2% (513)	7.2% (292)	4.7% (121)	7.9% (25)	0.0% (0)	5.8% (54)
2	5.8% (1373)	3.4% (289)	7.9% (565)	7.1% (288)	5.7% (146)	3.8% (12)	0.0% (0)	7.9% (73)
3	5.6% (1317)	4.8% (404)	5.6% (401)	4.6% (184)	10.4% (268)	4.8% (15)	0.0% (0)	4.8% (45)
4	10.9% (2573)	12.8% (1085)	8.8% (622)	6.0% (243)	19.0% (491)	12.4% (39)	0.0% (0)	10.0% (93)
Missing	72.3% (17024)	76.0% (6451)	70.4% (5006)	75.1% (3030)	60.3% (1557)	71.1% (224)	100% (92)	71.5% (664)
Basis of diagnosis								
Clinical	58.3% (13731)	66.5% (5644)	55.3% (3933)	49.6% (2004)	54.5% (1407)	34.9% (110)	0.0% (0)	68.1% (633)
Histology	37.2% (8765)	27.5% (2335)	41.4% (2942)	46.5% (1879)	44.1% (1138)	61.3% (193)	0.0% (0)	29.9% (278)
Death certificate	2.7% (635)	4.0% (342)	1.7% (118)	1.9% (76)	0.1% (2)	1.0% (3)	98.9% (91)	0.3% (3)
Not known	1.8% (424)	2.0% (171)	1.6% (114)	1.9% (78)	1.4% (36)	2.9% (9)	1.1% (1)	1.6% (15)

^a^Those that embarked, those with potential errors, those with missing vital status, and death certificate only (DCOs) excluded (*n* = 182).

### Patient characteristics and routes to diagnosis

The characteristics of cases varied by RtD (Table 1). People presenting via IP and OP were younger at diagnosis on average (66.0 and 66.4 years, respectively), and TWW and EP (71.9 and 70.2 years, respectively) older. After adjustment for other characteristics (Table 2), the odds of EP and TWW were higher for patients with older age at diagnosis and the odds of GP referral and OP lower. 78.1% of HCC cases were in men, and the proportion varied by RtD. After adjustment, the odds of an EP compared to any other route were 24% higher in women, and of a GP or TWW presentation 11 and 17% lower, respectively. Area-based deprivation was strongly associated with RtD; over 27% of EPs were in people in the most deprived quintile of the population and only 13.8% in the least deprived. A similar pattern was seen for GP and OP presentations, but not for TWW and IP presentations. After adjustment for other characteristics, odds of an EP presentation were 45% higher for those in the most deprived quintile compared to the least. In contrast, odds of OP presentation were 25% lower. The ethnicities of those diagnosed via EP, GP and OP were broadly similar, but a higher proportion of those diagnosed via TWW were white (87.2%), and for those diagnosed via IP a lower proportion were white (77.5%) and a higher proportion (6.7%) black. Ethnicity data was missing for a large proportion of DCOs (57%). After adjustment, there was no strong associations of ethnicity with EP, but odds of GP referral or TWW were highest for white people, and odds of OP were highest for Asian and other ethnicities.

**Table 2:** Associations of demographic, lifestyle, and clinical factors at HCC diagnosis with Route to Diagnosis.

	Emergency presentation				GP referral				Other outpatient				Two Week Wait			
	Univariate		Multivariate		Univariate		Multivariate		Univariate		Multivariate		Univariate		Multivariate	
	OR (95% CI)	*p*	OR (95% CI)	*p*	OR (95% CI)	*p*	OR (95% CI)	*p*	OR (95% CI)	*p*	OR (95% CI)	*p*	OR (95% CI)	*p*	OR (95% CI)	*p*
Diagnostic year	0.96 (0.95–0.97)	<0.001	0.96 (0.95–0.97)	<0.001	1.03 (1.02–1.04)	<0.001	1.02 (1.01–1.03)	<0.001	0.98 (0.97–0.99)	<0.001	0.97 (0.96–0.98)	<0.001	1.09 (1.08–1.11)	<0.001	1.11 (1.09–1.12)	<0.001
Age at diagnosis (per year)	1.01 (1.01–1.02)	<0.001	1.02 (1.02–1.02)	<0.001	0.99 (0.99–1.00)	<0.001	0.99 (0.99–1.00)	<0.001	0.98 (0.98–0.98)	<0.001	0.98 (0.98–0.98)	<0.001	1.02 (1.02–1.03)	<0.001	1.01 (1.00–1.01)	<0.001
Female gender	1.22 (1.14–1.30)	<0.001	1.24 (1.16–1.33)	<0.001	0.89 (0.83–0.95)	0.001	0.89 (0.83–0.96)	0.002	0.94 (0.87–1.02)	0.153	0.96 (0.88–1.05)	0.410	0.91 (0.82–1.00)	0.055	0.83 (0.75–0.92)	0.001
Deprivation quintile																
1-Least	1.00 (ref)		1.00 (ref)		1.00 (ref)		1.00 (ref)		1.00 (ref)		1.00 (ref)		1.00 (ref)		1.00 (ref)	
2	1.06 (0.97–1.17)	0.194	1.07 (0.97–1.19)	0.152	1.00 (0.91–1.10)	0.954	1 (0.91–1.10)	0.962	0.92 (0.82–1.03)	0.160	0.88 (0.78–0.99)	0.035	1.02 (0.89–1.17)	0.737	1.07 (0.93–1.24)	0.327
3	1.17 (1.07–1.28)	0.001	1.20 (1.09–1.32)	<0.001	0.97 (0.88–1.06)	0.507	0.96 (0.87–1.05)	0.351	0.95 (0.85–1.07)	0.392	0.89 (0.79–1.00)	0.053	0.98 (0.85–1.12)	0.717	1.06 (0.92–1.22)	0.403
4	1.30 (1.19–1.43)	<0.001	1.34 (1.22–1.48)	<0.001	0.88 (0.81–0.97)	0.009	0.88 (0.80–0.96)	0.006	0.96 (0.86–1.07)	0.490	0.86 (0.77–0.96)	0.010	0.90 (0.79–1.03)	0.130	1.06 (0.92–1.22)	0.429
5-Most	1.37 (1.25–1.49)	<0.001	1.45 (1.32–1.59)	<0.001	0.94 (0.86–1.03)	0.202	0.93 (0.85–1.02)	0.109	0.89 (0.80–0.99)	0.037	0.75 (0.67–0.84)	<0.001	0.77 (0.68–0.88)	<0.001	0.96 (0.84–1.11)	0.605
Ethnicity																
White	1.00 (ref)		1.00 (ref)		1.00 (ref)		1.00 (ref)		1.00 (ref)		1.00 (ref)		1.00 (ref)		1.00 (ref)	
Black	0.9 (0.76–1.06)	0.199	1.12 (0.94–1.34)	0.213	0.96 (0.81–1.13)	0.612	0.87 (0.72–1.04)	0.119	1.25 (1.04–1.52)	0.021	0.98 (0.79–1.20)	0.820	0.67 (0.50–0.89)	0.005	0.84 (0.62–1.14)	0.259
South Asian	1.11 (0.98–1.26)	0.090	1.13 (0.98–1.29)	0.083	0.99 (0.87–1.13)	0.919	0.95 (0.83–1.09)	0.489	1.15 (0.99–1.35)	0.074	0.98 (0.84–1.16)	0.853	0.60 (0.48–0.76)	<0.001	0.79 (0.62–1.01)	0.058
Other Asian	0.88 (0.73–1.05)	0.163	1.06 (0.87–1.29)	0.575	0.96 (0.79–1.15)	0.630	0.87 (0.71–1.06)	0.165	1.49 (1.22–1.83)	<0.001	1.25 (1.00–1.56)	0.046	0.70 (0.51–0.95)	0.022	0.8 (0.57–1.10)	0.171
Other ethnicity	1.06 (0.87–1.30)	0.565	1.15 (0.93–1.43)	0.192	0.75 (0.60–0.94)	0.013	0.71 (0.56–0.89)	0.003	1.58 (1.26–1.98)	<0.001	1.39 (1.10–1.77)	0.006	0.67 (0.47–0.96)	0.028	0.81 (0.56–1.18)	0.266
Not known	0.98 (0.87–1.11)	0.785	0.94 (0.82–1.07)	0.348	0.60 (0.52–0.70)	<0.001	0.66 (0.57–0.77)	<0.001	0.58 (0.48–0.70)	<0.001	0.76 (0.62–0.92)	0.005	0.88 (0.73–1.07)	0.213	0.64 (0.52–0.78)	<0.001
Primary liver disease																
Autoimmune hepatitis	0.67 (0.51–0.88)	0.004	0.66 (0.49–0.89)	0.006	1.46 (1.12–1.90)	0.006	1.41 (1.07–1.86)	0.014	1.58 (1.17–2.13)	0.003	1.50 (1.10–2.04)	0.010	0.29 (0.12–0.70)	0.006	0.29 (0.12–0.71)	0.007
Alcohol-related liver disease	1.00 (ref)		1.00 (ref)		1.00 (ref)		1.00 (ref)		1.00 (ref)		1.00 (ref)		1.00 (ref)		1.00 (ref)	
Haemochromatosis	0.43 (0.36–0.52)	<0.001	0.58 (0.47–0.70)	<0.001	1.48 (1.25–1.75)	<0.001	1.25 (1.05–1.48)	0.011	1.64 (1.36–1.97)	<0.001	1.64 (1.35–1.98)	<0.001	1.20 (0.89–1.62)	0.236	0.78 (0.57–1.06)	0.115
Hepatitis B	0.58 (0.49–0.68)	<0.001	0.78 (0.65–0.94)	0.007	1.19 (1.02–1.39)	0.029	1.13 (0.95–1.35)	0.165	1.74 (1.47–2.06)	<0.001	1.48 (1.22–1.79)	<0.001	0.79 (0.57–1.08)	0.141	0.68 (0.48–0.96)	0.030
Hepatitis C	0.65 (0.59–0.71)	<0.001	0.79 (0.71–0.88)	<0.001	1.29 (1.17–1.42)	<0.001	1.18 (1.07–1.31)	0.001	1.55 (1.39–1.73)	<0.001	1.35 (1.20–1.51)	<0.001	0.55 (0.44–0.68)	<0.001	0.51 (0.41–0.64)	<0.001
Non-alcoholic fatty liver disease	0.80 (0.73–0.88)	<0.001	0.80 (0.73–0.89)	<0.001	1.21 (1.10–1.33)	<0.001	1.16 (1.06–1.28)	0.002	0.90 (0.80–1.01)	0.080	0.96 (0.85–1.08)	0.458	1.56 (1.33–1.82)	<0.001	1.34 (1.14–1.57)	<0.001
Primary biliary cirrhosis	0.67 (0.55–0.82)	<0.001	0.60 (0.48–0.74)	<0.001	1.05 (0.86–1.29)	0.642	1.06 (0.85–1.31)	0.619	1.87 (1.52–2.31)	<0.001	1.91 (1.53–2.38)	<0.001	0.54 (0.33–0.87)	0.012	0.55 (0.33–0.90)	0.017
Other/unknown/none	0.78 (0.73–0.84)	<0.001	1.01 (0.92–1.12)	0.814	0.91 (0.85–0.98)	0.018	0.86 (0.78–0.95)	0.004	0.64 (0.59–0.71)	<0.001	0.84 (0.74–0.95)	0.005	2.78 (2.45–3.14)	<0.001	1.41 (1.20–1.64)	<0.001
Charlson score																
0	1.00 (ref)		1.00 (ref)		1.00 (ref)		1.00 (ref)		1.00 (ref)		1.00 (ref)		1.00 (ref)		1.00 (ref)	
1	0.96 (0.89–1.04)	0.345	0.97 (0.90–1.06)	0.533	1.16 (1.07–1.25)	<0.001	1.07 (0.98–1.16)	0.114	1.40 (1.27–1.55)	<0.001	1.38 (1.25–1.54)	<0.001	0.82 (0.74–0.92)	<0.001	0.80 (0.72–0.90)	<0.001
2	0.98 (0.90–1.07)	0.682	0.99 (0.90–1.09)	0.770	1.16 (1.06–1.28)	0.002	1.07 (0.97–1.18)	0.174	1.62 (1.45–1.82)	<0.001	1.73 (1.54–1.95)	<0.001	0.65 (0.56–0.74)	<0.001	0.57 (0.50–0.66)	<0.001
≥3	1.07 (1.00–1.14)	0.045	0.89 (0.83–0.96)	0.002	1.17 (1.10–1.25)	<0.001	1.14 (1.06–1.23)	<0.001	2.06 (1.90–2.23)	<0.001	2.08 (1.90–2.28)	<0.001	0.27 (0.24–0.30)	<0.001	0.32 (0.28–0.36)	<0.001
Cirrhosis																
None/NK	1.50 (1.41–1.61)	<0.001	1.15 (1.06–1.26)	0.001	0.65 (0.61–0.69)	<0.001	0.83 (0.76–0.90)	<0.001	0.51 (0.47–0.55)	<0.001	0.80 (0.72–0.89)	<0.001	2.16 (1.97–2.38)	<0.001	1.38 (1.22–1.57)	<0.001
Compensated	1.00 (ref)		1.00 (ref)		1.00 (ref)		1.00 (ref)		1.00 (ref)		1.00 (ref)		1.00 (ref)		1.00 (ref)	
Decompensated	4.15 (3.85–4.46)	<0.001	4.21 (3.91–4.54)	<0.001	0.42 (0.39–0.45)	<0.001	0.42 (0.39–0.46)	<0.001	0.57 (0.52–0.62)	<0.001	0.54 (0.49–0.59)	<0.001	0.43 (0.37–0.50)	<0.001	0.45 (0.39–0.53)	<0.001

Multivariate models were adjusted for age, gender, year of diagnosis, area-based deprivation quintile, ethnicity, Charlson comorbidity score, cirrhosis stage, and primary liver disease.

*OR* Odds Ratio, *CI* Confidence Interval.

Underlying PLD could be established from HES records in 13,777 (58.5%) cases. The remaining 41.5% either had one of the forms of PLD on our list but no record of a corresponding HES code for it, had a different PLD, or had no PLD. Overall, ALD (20.7% of cases) was the most common PLD, followed by NAFLD (15.1%) and then HCV (13.2%) and this varied by RtD. The odds of an EP presentation were highest for those with ALD compared to other PLDs, after adjustment for other characteristics. In comparison, the odds of a GP referral was higher for PLDs other than ALD. More people presenting via OP had a known PLD than those presenting via other routes. People with AIH, PBC, haemochromatosis, and viral hepatitis were more likely to have an OP presentation than those with ALD, or NAFLD. The odds of TWW presentation was highest in those with NAFLD and with no/unknown PLD.

41.7% of cases had no co-morbidity codes recorded in HES and 29.7% had a score of three or more. Those presenting via TWW or IP had fewest recorded comorbidities (57.2% and 55.9% had none, respectively) and those presenting via OP the most (52.0% had a score of two or more). After adjustment, those with known comorbidities were less likely to present via TWW (OR 0.32 [95%CI 0.28–0.36] in those with a score of ≥3 comorbidities compared to those with no recorded comorbidities), and more likely to present via OP (OR 2.08 [95%CI 1.90–2.28] for ≥3). The highest proportion of patients with decompensated cirrhosis was seen in those presenting via EP (accounting for 39.1% of all EPs). After adjustment, those with decompensated cirrhosis were over 4 times more likely to present via EP than those with compensated cirrhosis (OR 4.21 [95%CI 3.91–4.54]). TWW presentations were less likely to have any recorded cirrhosis. TNM stage was only available for 27.3% of cases. The stage distribution was more favourable in those presenting via GP or OP and least favourable for those presenting via EP.

### Routes to diagnosis over time

**Fig. 1:**
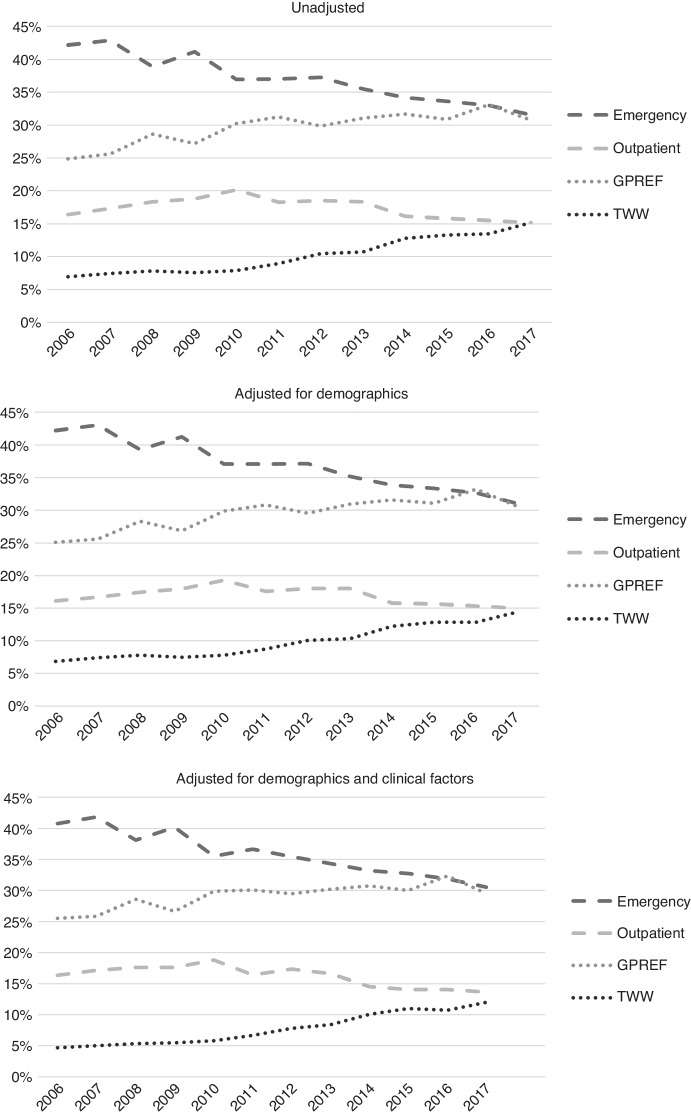
Proportion of HCC cases presenting via each main route to diagnosis, 2006–2017. Upper graph: unadjusted. Middle graph: adjusted for demographics. Lower graph: adjusted for demographic and clinical factors.

The proportion presenting via EP reduced between 2006 and 2017 from 42.2% to 31.5% of all cases, and the proportion presenting via GP referral and TWW increased (24.9% to 30.7% and 6.9% to 15.2%, respectively). There was no significant change in the proportion presenting via OP (16.4% to 15.2%). These patterns remained after adjustment for demographic and, additionally, for clinical characteristics of patients (Fig. 1).

### Associations with mortality

**Fig. 2:**
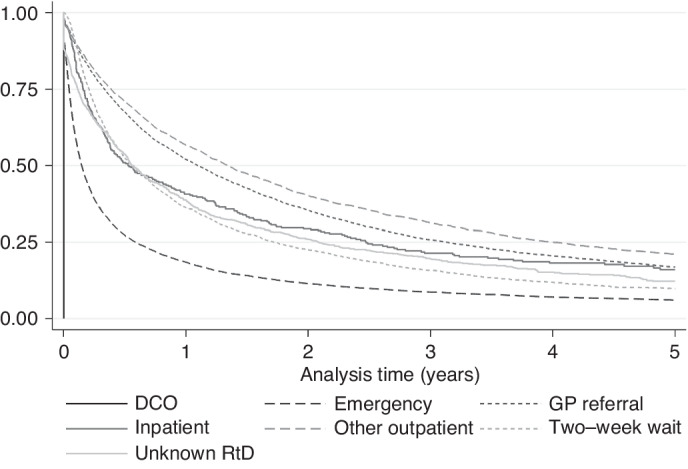
Kaplan–Meier curves. Kaplan–Meier 5-year survival by route to diagnosis.

20,671 (87.8%) HCC cases had died by the end of follow-up; median survival was 194 days overall (Table 1). Median survival ranged widely from 55 days for diagnoses via EP, to over 400 days for GP and OP (Fig. 2). Unadjusted, compared to EPs, those presenting via OP or GP referral had the lowest odds of mortality at 365 days (Table 3). In fully adjusted models, associations were only slightly attenuated and those presenting via OP or GP referral continued to have the lowest odds of mortality (OR 0.23 and 0.26, respectively) compared to EP. IP or TWW had intermediate odds (OR 0.37 and 0.42, respectively). In those with stage information, additional adjustment for TNM stage accounted for some of the variation in odds of mortality by RtD and further adjustment for receipt of potentially curative treatment attenuated associations further, but odds of death by 365 days post-diagnosis remained highest for EPs compared to other RtDs.

**Table 3:** Odds of 365-day mortality in HCC patients by route to diagnosis.

		Emergency	GP referral	Other outpatient	Two week wait	Inpatient elective	Unknown	*p* for difference
	*N*	OR	OR (95% CI)	OR (95% CI)	OR (95% CI)	OR (95% CI)	OR (95% CI)	*p*
Univariate	23,373	1.00 (ref)	0.21 (0.19–0.22)	0.17 (0.16–0.19)	0.40 (0.36–0.44)	0.33 (0.26–0.42)	0.36 (0.31–0.41)	<0.001
Full model^a^	**23,373**	**1.00 (ref)**	**0.26 (0.24**–**0.28)**	**0.23 (0.21**–**0.25)**	**0.42 (0.38**–**0.47)**	**0.37 (0.29**–**0.48)**	**0.39 (0.33**–**0.46)**	**<0.001**
Sensitivity Analyses								
Full model, those with known TNM stage only	6514	1.00 (ref)	0.28 (0.24–0.33)	0.23 (0.20–0.28)	0.56 (0.47–0.67)	0.40 (0.25–0.63)	0.33 (0.25–0.44)	<0.001
Full model with adjustment for TNM stage	6514	1.00 (ref)	0.37 (0.32–0.44)	0.32 (0.26–0.39)	0.56 (0.46–0.69)	0.49 (0.28–0.83)	0.39 (0.28–0.54)	<0.001
Full model with adjustment for TNM stage & curative treatment	6514	1.00 (ref)	0.41 (0.35–0.49)	0.40 (0.32–0.49)	0.62 (0.50–0.77)	0.51 (0.28–0.92)	0.38 (0.27–0.54)	<0.001
Stratified by Cirrhosis stage								
None/not known	9946	1.00 (ref)	0.22 (0.19–0.25)	0.19 (0.16–0.22)	0.35 (0.31–0.41)	0.33 (0.23–0.48)	0.37 (0.30–0.47)	<0.001
Compensated	7642	1.00 (ref)	0.28 (0.25–0.32)	0.24 (0.21–0.28)	0.47 (0.38–0.56)	0.32 (0.21–0.51)	0.34 (0.26–0.44)	<0.001
Decompensated	5785	1.00 (ref)	0.34 (0.29–0.40)	0.32 (0.26–0.38)	0.95 (0.62–1.46)	0.51 (0.28–0.94)	0.54 (0.34–0.85)	<0.001
Stratified by Primary liver disease^b^								
Autoimmune hepatitis or primary biliary cirrhosis	718	1.00 (ref)	0.26 (0.17–0.40)	0.27 (0.17–0.42)	0.44 (0.17–1.11)	0.12 (0.01–1.28)	0.19 (0.07–0.52)	<0.001
Alcohol-related liver disease	4856	1.00 (ref)	0.31 (0.26–0.36)	0.27 (0.23–0.32)	0.59 (0.46–0.77)	0.52 (0.30–0.91)	0.46 (0.32–0.66)	<0.001
Haemochromatosis	672	1.00 (ref)	0.21 (0.13–0.34)	0.27 (0.16–0.46)	0.86 (0.42–1.76)	0.56 (0.14–2.32)	0.43 (0.16–1.19)	<0.001
Hepatitis B	841	1.00 (ref)	0.25 (0.17–0.38)	0.17 (0.11–0.26)	0.45 (0.23–0.90)	0.47 (0.17–1.33)	0.28 (0.11–0.71)	<0.001
Hepatitis C	3092	1.00 (ref)	0.37 (0.31–0.45)	0.28 (0.23–0.35)	0.75 (0.50–1.13)	0.44 (0.21–0.91)	0.48 (0.31–0.73)	<0.001
Non-alcoholic fatty liver disease	3545	1.00 (ref)	0.25 (0.21–0.31)	0.24 (0.19–0.31)	0.35 (0.27–0.47)	0.22 (0.11–0.46)	0.20 (0.12–0.34)	<0.001
Other/unknown/none	9649	1.00 (ref)	0.22 (0.19–0.25)	0.19 (0.16–0.23)	0.34 (0.29–0.39)	0.33 (0.23–0.48)	0.48 (0.38–0.60)	<0.001
Stratified by age								
<65 years	7730	1.00 (ref)	0.33 (0.29–0.37)	0.28 (0.24–0.32)	0.49 (0.40–0.60)	0.42 (0.29–0.62)	0.48 (0.37–0.61)	<0.001
≥65 years	15,643	1.00 (ref)	0.24 (0.21–0.26)	0.21 (0.19–0.23)	0.40 (0.35–0.45)	0.35 (0.25–0.49)	0.35 (0.28–0.43)	<0.001

*OR* Odds Ratio, *CI* Confidence Interval.

Bold represents main results (the full model).

^a^Adjusted for age, gender, year of diagnosis, deprivation quintile, ethnicity, Charlson score, cirrhosis, and underlying primary liver disease.

^b^Ethnicity removed due to low numbers in groups.

In the sensitivity analyses (Table 3), for people with decompensated cirrhosis, odds of mortality by 365 days were similar for those diagnosed via TWW and EP, and lowest for GP and OP. Odds of 365-day mortality were highest for EPs irrespective of the underlying cause of PLD, although odds were also high for presentations via TWW for people with haemochromatosis and HCV. For those with AIH, PBC or NAFLD, the lowest odds of 365-day mortality were diagnoses via IP or Unknown RtDs, followed by GP and OP. Analyses were repeated with 90-day mortality as the outcome to assess if associations were different for short term mortality (Supplementary Information 5). Associations were broadly similar.

### Associations of RtD with curative treatment

20.5% of cases received potentially curative treatment overall, which also varied widely by RtD (Table 1); from 9.0% for EP to 35.8% for OP. Unadjusted, compared to EP, the odds of curative treatment were almost six times higher for OP (OR 5.94), four times higher for GP and IP (ORs 3.90 and 3.86, respectively) and two times higher for TWW (OR 1.91), and Unknown RtD (OR 1.92) (Table 4). In the fully adjusted model, these associations remained but were slightly attenuated (for example, OR for OP was 4.16 compared to EP). In sensitivity analyses, variations in associations were seen. Excluding those that died in the first 90 days after diagnosis attenuated associations substantially, though odds of curative treatment were still 1.8 to 2.3 times higher for presentations via OP, IP, and GP compared to EP. The same attenuation pattern was also seen following additional adjustment for TNM stage. When stratified by cirrhosis stage, large differences in odds of curative treatment by RtD were seen for those with no known cirrhosis (OR range 6.05 for OP to 1.0 for EP (referent)), and compensated cirrhosis (OR range 4.41 for OP to 1.0 for EP). For those with decompensated cirrhosis, odds were highest in those that presented via OP (OR 3.05) and GP (OR 2.20), but those that presented via TWW, IP or unknown RtD all had similar odds to those presenting via EP. Some differences in range of associations were also seen by PLD. For those with haemochromatosis or HBV, odds of curative treatment in those presenting via OP were over six times that of those presenting via EP. For those with NAFLD, odds of curative treatment were 4–5 times higher in those presenting via IP, OP, or GP referral than via EP. For those with other/unknown/no PLD, odds were much higher for OP (OR 6.01) and GP (OR 4.60), compared to EP. An effect modification by age was also seen; those aged over 65 were over five times more likely to receive curative treatment if presentation was via OP than EP, whereas those under 65 were three times more likely.

**Table 4:** Odds of curative treatment in HCC patients by route to diagnosis.

		Emergency	GP referral	Other outpatient	Two week wait	Inpatient elective	Unknown	*p* for difference
	*N*	OR	OR (95% CI)	OR (95% CI)	OR (95% CI)	OR (95% CI)	OR (95% CI)	
Univariate	23,463	1.00 (ref)	3.90 (3.56–4.27)	5.64 (5.11–6.22)	1.91 (1.68–2.17)	3.86 (2.98–5.00)	1.92 (1.58–2.32)	<0.001
Full model^a^	**23,460**	**1.00 (ref)**	**3.21 (2.92**–**3.53)**	**4.16 (3.75**–**4.62)**	**2.10 (1.83**–**2.40)**	**3.28 (2.49**–**4.32)**	**1.77 (1.44**–**2.16)**	**<0.001**
Sensitivity Analyses								
Full model. excluding those that died in the first 90 days	14,959	1.00 (ref)	1.79 (1.61–1.99)	2.29 (2.05–2.57)	1.22 (1.05–1.41)	2.17 (1.60–2.95)	1.07 (0.86–1.32)	<0.001
Full model, those with TNM stage only	6,531	1.00 (ref)	3.15 (2.64–3.76)	4.32 (3.54–5.27)	1.75 (1.39–2.21)	2.68 (1.62–4.44)	2.10 (1.51–2.93)	<0.001
Full model plus adjustment for TNM stage	6,531	1.00 (ref)	2.18 (1.78–2.66)	2.94 (2.34–3.68)	1.78 (1.36–2.31)	2.03 (1.13–3.62)	1.50 (1.04–2.18)	<0.001
Stratified by Cirrhosis stage								
None/not known	10,002	1.00 (ref)	4.88 (4.07–5.85)	6.05 (4.95–7.39)	3.21 (2.60–3.96)	3.96 (2.56–6.13)	1.96 (1.41–2.73)	<0.001
Compensated	7,657	1.00 (ref)	2.84 (2.44–3.31)	3.73 (3.17–4.40)	1.66 (1.31–2.11)	4.41 (2.82–6.90)	1.95 (1.44–2.65)	<0.001
Decompensated	5,801	1.00 (ref)	2.20 (1.82–2.67)	3.05 (2.50–3.73)	0.77 (0.42–1.42)	1.57 (0.74–3.33)	1.48 (0.85–2.55)	<0.001
Stratified by primary liver disease								
Autoimmune hepatitis or primary biliary cirrhosis	719	1.00 (ref)	1.96 (1.20–3.20)	2.10 (1.28–3.44)	0.89 (0.24–3.24)	5.21 (0.59–46.02)	1.56 (0.55–4.39)	0.0324
Alcohol-related liver disease	4,861	1.00 (ref)	2.70 (2.23–3.27)	3.23 (2.62–3.97)	1.74 (1.25–2.42)	2.36 (1.27–4.41)	1.45 (0.91–2.33)	<0.001
Haemochromatosis	672	1.00 (ref)	4.90 (2.74–8.76)	6.21 (3.38–11.40)	1.53 (0.64–3.65)	3.17 (0.69–14.55)	0.63 (0.13–3.08)	<0.001
Hepatitis B	847	1.00 (ref)	3.84 (2.45–6.02)	6.86 (4.33–10.87)	1.88 (0.85–4.14)	2.18 (0.74–6.43)	3.12 (1.19–8.16)	<0.001
Hepatitis C	3,103	1.00 (ref)	2.00 (1.63–2.47)	2.93 (2.36–3.65)	0.91 (0.56–1.49)	4.49 (2.21–9.12)	1.31 (0.83–2.08)	<0.001
Non-alcoholic fatty liver disease	3,554	1.00 (ref)	4.02 (3.08–5.24)	4.70 (3.50–6.32)	2.49 (1.75–3.56)	5.28 (2.55–10.92)	3.64 (2.12–6.25)	<0.001
Other/unknown/none	9,704	1.00 (ref)	4.60 (3.79–5.58)	6.01 (4.85–7.45)	3.25 (2.61–4.05)	3.40 (2.10–5.49)	1.66 (1.18–2.34)	<0.001
Stratified by age								
<65 years	7,777	1.00 (ref)	2.33 (2.03–2.68)	3.10 (2.67–3.59)	1.72 (1.37–2.17)	2.64 (1.80–3.87)	1.41 (1.07–1.87)	<0.001
≥65 years	15,683	1.00 (ref)	4.11 (3.58–4.72)	5.36 (4.61–6.24)	2.41 (2.01–2.89)	4.04 (2.72–6.00)	2.13 (1.59–2.85)	<0.001

*OR* Odds Ratio, *CI* Confidence Interval.

Bold represents main results (the full model).

^a^Adjusted for age, gender, year of diagnosis, deprivation quintile, ethnicity, Charlson score, cirrhosis, and underlying primary liver disease.

## Discussion

### Summary

Route to diagnosis is strongly associated with odds of 90-day and 365-day mortality and inversely associated with receipt of potentially curative treatment in patients diagnosed with HCC. Patients presenting via EP had the poorest prognosis; <10% received curative treatment and median survival was just 55 days. In comparison, of those presenting via OP, over a third received curative treatment, and median survival was nearly10 times higher than for EP diagnoses. After adjustment for demographic and clinical factors, odds of mortality by 365 days was substantially lower for those presenting via GP or OP routes compared to EP, and odds of curative treatment substantially higher. The differences in rates of potentially curative treatment by RtD were influenced by differences in patient age at diagnosis, PLD, cancer and cirrhosis stage at presentation, and early death. Vulnerable patients including older people, women, those with alcohol-related liver disease, and people residing in more deprived areas, were at increased risk of an EP. Fortunately, the proportion of HCC patients presenting as emergencies is decreasing, and those presenting via GP referrals increasing.

### Strengths and limitations

This study used a population-based dataset including the vast majority of HCC cases diagnosed and treated in England. The results are therefore representative of and generalisable to the population. 12 years of data were included which allowed examination of differences over time. The sample size was large (*n* = 23,555), despite liver cancer being classified as a rare cancer. RtD is a well-established metric derived from linked routinely-collected population-based datasets. We were able to examine associations of RtD with a wide range of factors known to cause variation in mortality available within and derived from the high-quality National Cancer Registration Dataset. We present a novel method for identification of primary liver cancer from HES codes, validated by comparison with PLD from clinical records.

There are limitations. As data was not collected specifically for the purpose of this study, in common with other observation studies based on routine data, some variables that would have been informative were not available in the dataset. In particular, Barcelona liver cancer stage or variables to derive this (TNM stage, Child-Pugh liver function and ECOG performance status) were not available for the majority of cases. Some important liver cancer risk factors such as BMI, smoking and alcohol consumption, or indicators of liver function, were not available. In our study, underlying cause of primary liver cancer, comorbidity score, and cirrhosis stage were inferred from HES codes, which are dependent on hospital admissions and accurate coding practices. PLD and cirrhosis status were not derivable for around 40% of patients, and it was not possible to know if this was true absence of a PLD or just absence of a code. Furthermore, we used data to identify underlying PLD both before and after the diagnosis of HCC. This was done to improve classification recognising that this is an understudied area, but there is the risk of bias through differential misclassification by RtD. Liver surveillance status was unknown. The deprivation measure used was based on area of residence, therefore there may be residual confounding by socioeconomic status. There were insufficient numbers of inpatient presentations to assess associations with this RtD metric. In addition, we were unable to use cause of death to identify cancer-related death due to the propensity to assign deaths in people with HCC as cancer-related although many would have been due to complications of cirrhosis (77% of deaths were attributed to C220 in our cohort).

### Comparison with other literature

For all cancers diagnosed 2011 to 2015 combined, screening followed by TWW and then GP referral had the best 12-month net survival, but for liver cancer the other outpatient route had the best survival [18]. (Note no liver cancers were diagnosed via the screening route as there is not yet a national liver cancer screening programme, although surveillance of at-risk groups is advised). Similar to our study, excess early mortality (the three months after diagnosis) in emergency presentations has previously been reported for colorectal, cervical, breast, lung, and prostate, even after adjustment for age, stage and comorbidity [19]. A reduction in emergency presentations across all cancers from 2006–2013 was previously reported, of which only one-fifth was explained by change in case mix (age, gender, deprivation and cancer site) [20]. Our results show change in case-mix had very little influence on these RtD differences over time in those with liver cancer. Similar to our findings for liver cancer, Tataru et al. [21] found that patients with lung cancer were much less likely to receive surgery if they presented as an emergency. Markar et al. [22] found that oesophageal and gastric cancer patients that had an emergency diagnosis had poorer prognosis after surgery. Both of these studies, and another large study, including liver cancer, reported female gender, increased age, and higher deprivation to be risk factors for emergency presentations [21–23].

### Interpretation

In order to improve cancer survival in England, the Independent Cancer Taskforce has identified early diagnosis as key [4]. The high rate of EPs in those with decompensated cirrhosis likely reflects the development of significant symptoms requiring admission (e.g. variceal bleeding/ ascites). The HCC may be diagnosed as part of the investigation of those symptoms. For liver cancer, identifying at-risk patients (i.e. those with asymptomatic compensated cirrhosis or chronic hepatitis B) and closely monitoring them may reduce the number of emergency presentations and improve early diagnosis. Those diagnosed via outpatient routes were most likely to survive for one year after diagnosis and receive potentially curative treatments. People with viral or autoimmune hepatitis, or multimorbidity, were more likely to be diagnosed via OP. These patients would likely have received regular specialist medical care for their chronic liver conditions, which may have led to earlier cancer detection. Similar to OP, patients presenting via GP referral may be interacting regularly with healthcare, thus their more favourable cancer and cirrhosis stage profile at diagnosis. We were unable to assess the impact of surveillance, but 6-monthly liver ultrasounds, with or without serum alpha-fetoprotein testing, have been shown to improve early detection, receipt of curative treatment and survival [7] and is therefore recommended for people with cirrhosis [8]. Patients with known cirrhosis undergoing liver surveillance would be most likely to be categorised as OP presentations. Early detection of HCC in patients with NAFLD, the most rapidly increasing cause of HCC [24], via ultrasound can be challenging [25]. There is also the challenge of educating patients and clinicians to identify signs and symptoms of HCC, and to increase referrals for diagnostic investigation. A small study in Scotland of GP interactions prior to a cancer diagnosis found not having seen a GP was a strong risk factor of an EP, though 18% of EPs in upper GI cancer cases were missed opportunities (GPs had not adhered to referral guidelines for suspected cancers) [26]. Of these, over 70% had seen their GP 6 times or more time in the 24 months preceding a diagnosis. A similar proportion occurred while patients were waiting for secondary care appointments.

### Conclusions

Rates of liver cancer have been rapidly increasing in the UK and survival is still poor, making liver cancer one of the most rapidly increasing causes of cancer-related death in the UK. Identifying factors associated with treatment and mortality can help identify areas to target to improve early diagnosis and survival. Liver cancer patients are most likely to present via the emergency route to diagnosis, which is strongly associated with higher mortality in the first year after diagnosis and reduced odds of curative treatment, even after adjustment for early mortality and prognostic factors. There was inequality among the cases, where older patients, women and those from more deprived areas are most likely to present as emergencies. The trend toward decreasing emergency presentations and increasing GP referrals needs to continue. Ensuring patients at higher risk of HCC, including those with high BMIs, are identified and regularly monitored, and that both patients and health care professionals are aware of liver cancer risk factors and symptoms, may help increase diagnoses at a curable stage.

### Supplementary information

Supplementary information

## Data Availability

Access to cancer registration data through NHS England can be granted provided there is a justified purpose for the data release, and that there is an appropriate legal basis with safeguards in place to protect the data. Dependent on the request, ethical approval may be required. The access process is managed by the Data Access Request Service.
