# Supplementary material for: Routes to diagnosis for hepatocellular carcinoma patients: predictors and associations with treatment and mortality
Source: Br J Cancer. 2024 Mar 18;130(10):1697–708. doi: 10.1038/s41416-024-02645-3 (PMC11091115; doi:10.1038/s41416-024-02645-3)
Supplement: Supplementary file 1 — Supplementary information [file 41416_2024_2645_MOESM1_ESM.docx]

**Supplementary information**

SI1: Cirrhosis and decompensation codes used to derive cirrhosis status

| Code | Description | Cirrhosis | Decompensated cirrhosis* |
| --- | --- | --- | --- |
| ICD10 |  |  |  |
| K70.3 | Alcoholic cirrhosis of liver | Y |  |
| K71.7 | Toxic liver disease with fibrosis and cirrhosis of liver | Y |  |
| K72.1 | Chronic hepatic failure | Y |  |
| K72.9 | Hepatic failure, unspecified | Y |  |
| K74.4 | Secondary biliary cirrhosis | Y |  |
| K74.5 | Biliary cirrhosis, unspecified | Y |  |
| K74.6 | Other and unspecified cirrhosis of liver | Y |  |
| K76.6 | Portal hypertension | Y |  |
| I85.0 | Oesophageal varices | Y | Y |
| I85.9 | Oesophageal varices without bleeding | Y |  |
| I86.4 | Gastric varices | Y | Y |
| I98.2 | Oesophageal varices without bleeding in diseases classified elsewhere | Y | Y |
| I98.3 | Oesophageal varices with bleeding in diseases classified elsewhere | Y | Y |
| K70.4 | Alcoholic hepatic failure | Y |  |
| K70.9 | Alcoholic liver disease, unspecified | Y |  |
| R18.X | Ascites | Y (pre HCC only) | Y |
| K92.0 | Hematemesis |  | Y |
| K92.1 | Melena |  | Y |
| K92.2 | Gastrointestinal haemorrhage |  | Y |
| OPCS4 |  |  |  |
| T46 | Treatments of Ascites | Y (pre HCC only) | Y |
| G10.4 | Local ligation of varices of oesophagus | Y | Y |
| G10.8 | Other specified open operations on varices of oesophagus | Y | Y |
| G10.9 | Unspecified open operations on varices of oesophagus | Y | Y |
| G14.4 | Fibreoptic endoscopic injection sclerotherapy to varices of oesophagus | Y | Y |
| G17.4 | Endoscopic injection sclerotherapy to varices of oesophagus using rigid oesophagoscope | Y | Y |
| G43.4 | Fibreoptic endoscopic sclerotherapy to lesion of upper gastrointestinal tract | Y | Y |
| G43.7 | Fibreoptic endoscopic rubber band ligation of upper gastrointestinal tract varices | Y | Y |
| J06.1 | Transjugular intrahepatic insertion of stent into portal vein | Y | Y |
| J06.2 | Transjugular intrahepatic insertion of stent graft into portal vein | Y | Y |

based on the method described in Driver et al ^1^. *Up to 60 days post diagnosis. Y=Yes

SI2: Codes used to derive underlying primary liver disease (PLD)

| Categories of liver disease: | Code (ICD10) | Description |
| --- | --- | --- |
| Hepatitis C | B18.2 | Chronic viral hepatitis C |
| Hepatitis B | B18.0  B18.1 | Chronic viral hepatitis B with delta-agent  Chronic viral hepatitis B without delta-agent |
| Primary Biliary Cirrhosis (PBC) | K74.3 | Primary biliary cirrhosis |
| Autoimmune Hepatitis (AIH) | K73.2  K75.4 | Chronic active hepatitis, not elsewhere classified  Autoimmune hepatitis |
| Haemochromatosis | E83.1 | Disorders of iron metabolism |
| Alcohol-related liver disease (ALD) | F10  K70 | Mental and behavioural disorders due to use of alcohol  Alcoholic liver disease |
| Non-alcoholic Fatty Liver (NAFLD) | K76.0, or  Cirrhosis* &  (DBM  E10  E11  E13  E14  or Obesity E66)  & no other PLD | Fatty (change of) liver, not elsewhere classified  Type 1 diabetes mellitus  Type 2 diabetes mellitus  Other specified diabetes mellitus  Unspecified diabetes mellitus  Obesity |
| Other/Unknown | Null | None of the specified PLD codes |

* Cirrhosis as defined in SI1 above and Driver et al ^1^

SI3: Performance of the PLD algorithm in the Leeds Teaching Hospitals NHS Trust dataset, 2007-2016, with increasing length of follow-up for inclusion of inpatient episode codes

|  | **Algorithm** | |
| --- | --- | --- |
| **Time after HCC Diagnosis/ days** | Correct PLD | Κappa-statistic |
| 0* | 60% | 0.47 |
| 30 | 63% | 0.52 |
| 60 | 66% | 0.57 |
| 90 | 71% | 0.62 |
| 120 | 72% | 0.64 |
| 180 | 73% | 0.65 |
| 365 | 74% | 0.67 |

Κappa statistic for overall agreement. *only codes pre-HCC diagnosis included.

SI4: Performance of primary liver disease algorithm in the Leeds Teaching Hospitals NHS Trust dataset, 2007-2016

|  | | **PLD Predicted by Algorithm** | | | | | | | **Total** | **Correct Aetiology** |
| --- | --- | --- | --- | --- | --- | --- | --- | --- | --- | --- |
|  |  | Other | HCV | HBV | PBC | Haemo | ALD | NAFLD |  |  |
| **True PLD** | Other | 71 | 0 | 0 | 0 | 1 | 8 | 8 | 88 | 81% |
|  | HCV | 5 | 37 | 0 | 0 | 0 | 2 | 0 | 44 | 84% |
|  | HBV | 4 | 2 | 10 | 0 | 0 | 1 | 0 | 17 | 59% |
|  | PBC | 0 | 0 | 0 | 7 | 0 | 0 | 0 | 7 | 100% |
|  | Haemo | 3 | 0 | 0 | 0 | 15 | 1 | 0 | 19 | 79% |
|  | ALD | 9 | 0 | 0 | 0 | 1 | 54 | 4 | 68 | 79% |
|  | NAFLD | 20 | 1 | 0 | 0 | 2 | 0 | 20 | 43 | 47% |
|  | Total | 112 | 40 | 10 | 7 | 19 | 66 | 32 | 286 | 75% |

Agreement (shaded cells) between primary liver disease PLD according to algorithm with one year of follow-up and true PLD according to clinical records. HCV = hepatitis C, HBV = hepatitis B, PBC = primary biliary cirrhosis, Haemo = haemochomatosis, ALD = alcohol-related liver disease, NAFLD = non-alcoholic fatty liver disease. Too few autoimmune hepatitis cases to assess, therefore these were excluded (n=3). Note, NAFLD algorithm did not include obesity

SI5: Odds of 365-day mortality in HCC patients by Route to Diagnosis

|  |  | **Emergency** | **GP referral** | **Other outpatient** | **Two week wait** | **Inpatient elective** | **Unknown** | **p for difference** |
| --- | --- | --- | --- | --- | --- | --- | --- | --- |
|  | **N** | **OR** | **OR (95% CI)** | **OR (95% CI)** | **OR (95% CI)** | **OR (95% CI)** | **OR (95% CI)** |  |
| Univariate | 23,373 | 1.00 (ref) | 0.17 (0.15 - 0.18) | 0.15 (0.13 - 0.16) | 0.27 (0.25 - 0.30) | 0.34 (0.27 - 0.43) | 0.35 (0.30 - 0.40) | <0.001 |
| **Full model*** | **23,373** | **1.00 (ref)** | **0.21 (0.19 - 0.22)** | **0.19 (0.18 - 0.21)** | **0.29 (0.26 - 0.32)** | **0.36 (0.28 - 0.46)** | **0.37 (0.31 - 0.43)** | **<0.001** |
| Sensitivity Analyses |  |  |  |  |  |  |  |  |
| Full model, those with TNM stage only | 6,514 | 1.00 (ref) | 0.21 (0.18 - 0.25) | 0.22 (0.18 - 0.26) | 0.41 (0.35 - 0.49) | 0.37 (0.22 - 0.61) | 0.32 (0.24 - 0.45) | <0.001 |
| Full model with adjustment for TNM | 6,514 | 1.00 (ref) | 0.26 (0.22 - 0.31) | 0.29 (0.23 - 0.36) | 0.41 (0.34 - 0.49) | 0.42 (0.24 - 0.73) | 0.37 (0.26 - 0.53) | <0.001 |
| Full model with adjustment for TNM & curative treatment | 6,514 | 1.00 (ref) | 0.28 (0.24 - 0.33) | 0.33 (0.27 - 0.42) | 0.43 (0.36 - 0.52) | 0.43 (0.24 - 0.76) | 0.38 (0.27 - 0.54) | <0.001 |
| Stratified by Cirrhosis stage |  |  |  |  |  |  |  |  |
| None/not known | 9,946 | 1.00 (ref) | 0.21 (0.19 - 0.24) | 0.23 (0.19 - 0.26) | 0.27 (0.24 - 0.30) | 0.32 (0.22 - 0.46) | 0.43 (0.35 - 0.52) | <0.001 |
| Compensated | 7,642 | 1.00 (ref) | 0.14 (0.12 - 0.16) | 0.13 (0.11 - 0.16) | 0.27 (0.21 - 0.34) | 0.30 (0.17 - 0.53) | 0.20 (0.14 - 0.29) | <0.001 |
| Decompensated | 5,785 | 1.00 (ref) | 0.29 (0.25 - 0.34) | 0.24 (0.20 - 0.28) | 0.58 (0.43 - 0.78) | 0.50 (0.30 - 0.83) | 0.38 (0.25 - 0.58) | <0.001 |
| Stratified by PLD** |  |  |  |  |  |  |  |  |
| Autoimmune hepatitis or primary biliary cirrhosis | 718 | 1.00 (ref) | 0.17 (0.11 - 0.27) | 0.12 (0.07 - 0.20) | 0.19 (0.06 - 0.61) | 0.30 (0.03 - 3.34) | 0.10 (0.02 - 0.46) | <0.001 |
| Alcohol-related liver disease | 4,856 | 1.00 (ref) | 0.22 (0.19 - 0.26) | 0.19 (0.16 - 0.23) | 0.39 (0.30 - 0.51) | 0.43 (0.24 - 0.76) | 0.33 (0.23 - 0.49) | <0.001 |
| Haemochromatosis | 672 | 1.00 (ref) | 0.21 (0.13 - 0.36) | 0.30 (0.17 - 0.52) | 0.35 (0.16 - 0.80) | 0.52 (0.10 - 2.72) | 0.21 (0.05 - 0.81) | <0.001 |
| Hepatitis B | 841 | 1.00 (ref) | 0.13 (0.08 - 0.21) | 0.11 (0.07 - 0.20) | 0.42 (0.20 - 0.88) | 0.31 (0.09 - 1.04) | 0.38 (0.14 - 1.06) | <0.001 |
| Hepatitis C | 3,092 | 1.00 (ref) | 0.20 (0.16 - 0.26) | 0.15 (0.11 - 0.20) | 0.44 (0.28 - 0.71) | 0.49 (0.22 - 1.12) | 0.35 (0.20 - 0.59) | <0.001 |
| Non-alcoholic fatty liver disease | 3,545 | 1.00 (ref) | 0.18 (0.15 - 0.22) | 0.19 (0.14 - 0.24) | 0.25 (0.19 - 0.34) | 0.29 (0.14 - 0.60) | 0.17 (0.09 - 0.33) | <0.001 |
| Other/unknown/none | 9,649 | 1.00 (ref) | 0.23 (0.20 - 0.26) | 0.24 (0.21 - 0.28) | 0.28 (0.25 - 0.32) | 0.36 (0.25 - 0.51) | 0.56 (0.46 - 0.68) | <0.001 |
| Stratified by age |  |  |  |  |  |  |  |  |
| <65 years | 7,730 | 1.00 (ref) | 0.22 (0.19 - 0.26) | 0.20 (0.17 - 0.23) | 0.34 (0.27 - 0.42) | 0.36 (0.24 - 0.55) | 0.38 (0.29 - 0.49) | <0.001 |
| ≥65 years | 15,643 | 1.00 (ref) | 0.20 (0.18 - 0.22) | 0.20 (0.18 - 0.22) | 0.28 (0.25 - 0.32) | 0.37 (0.27 - 0.51) | 0.36 (0.30 - 0.44) | <0.001 |

* Adjusted for age, gender, year of diagnosis, deprivation quintile, ethnicity, Charlson score, cirrhosis, and underlying PLD

** Ethnicity removed due to low numbers in groups

OR Odds Ratio, CI Confidence Interval

**References**

1. Driver RJ, Balachandrakumar V, Burton A, et al. Validation of an algorithm using inpatient electronic health records to determine the presence and severity of cirrhosis in patients with hepatocellular carcinoma in England: an observational study. *BMJ Open* 2019;9(7):e028571. doi: 10.1136/bmjopen-2018-028571
